# The anti-adhesive mode of action of a purified mushroom (*Lentinus edodes*) extract with anticaries and antigingivitis properties in two oral bacterial phatogens

**DOI:** 10.1186/1472-6882-14-75

**Published:** 2014-02-24

**Authors:** Caterina Signoretto, Anna Marchi, Anna Bertoncelli, Gloria Burlacchini, Adele Papetti, Carla Pruzzo, Egija Zaura, Peter Lingström, Itzhak Ofek, Jonathan Pratten, David A Spratt, Michael Wilson, Pietro Canepari

**Affiliations:** 1 Dipartimento di Patologia e Diagnostica – Sezione di Microbiologia, Università di Verona, Strada Le Grazie 8, 37134 Verona, Italy; 2 Dipartimento di Scienze del Farmaco, Università di Pavia, Via Taramelli 12, 27100 Pavia, Italy; 3 DISTAV, Università di Genova, Corso Europa 26, 16132 Genova, Italy; 4 Department of Preventive Dentistry, Academic Centre for Dentistry Amsterdam (ACTA), Gustav Mahlerlaan 3004, 1081, LA, Amsterdam, the Netherlands; 5 Department of Cariology, Institute of Odontology at Sahlgrenska Academy, University of Gothenburg, Box 450, 405 30 Göteborg, Sweden; 6 Department of Clinical Microbiology and Immunology, Sackler Faculty of Medicine, Tel Aviv University, 39987 Tel Aviv, Israel; 7 Department of Microbial Diseases, UCL Eastman Dental Institute, 256 Gray’s Inn Road, London WC1X 8LD, UK

**Keywords:** *Streptococcus mutans*, *Prevotella intermedia*, Dental caries, Gingivitis, Mushroom extract

## Abstract

**Background:**

In previous works we have shown that a low-molecular-mass (LMM) fraction from mushroom (*Lentinus edodes*) homogenate interferes with binding of *Streptococcus mutans* to hydroxyapatite and *Prevotella intermedia* to gingival cells. Additionally, inhibition of biofilm formation of both odonto- and periodonto-pathogenic bacteria and detachment from preformed biofilms have been described for this compound. Further purification of mushroom extract has been recently achieved and a sub-fraction (i.e. # 5) has been identified as containing the majority of the mentioned biological activities. The aim of this study was to characterise the bacterial receptors for the purified mushroom sub-fraction #5 in order to better elucidate the mode of action of this compound when interfering with bacterial adhesion to host surfaces or with bacteria-bacteria interactions in the biofilm state.

**Methods:**

Candidate bacterial molecules to act as target of this compound were bacterial surface molecules involved in cell adhesion and biofilm formation, and, thus, we have considered cell wall associated proteins (CWPs), teichoic acid (TA) and lipoteichoic acid (LTA) of *S. mutans*, and outer membrane proteins (OMPs) and lipopolysaccharide (LPS) of *P. intermedia*.

**Results:**

Fifteen *S. mutans* CWPs and TA were capable of binding sub-fraction #5, while LTA did not. As far as *P. intermedia* is concerned, we show that five OMPs interact with sub-fraction # 5. Capacity of binding to *P. intermedia* LPS was also studied but in this case negative results were obtained.

**Conclusions:**

Binding sub-fraction # 5 to surface molecules of *S. mutans* or *P. intermedia* may result in inactivation of their physiological functions. As a whole, these results indicate, at molecular level, the bacterial surface alterations affecting adhesion and biofim formation. For these antimicrobial properties, the compound may find use in daily oral hygiene.

## Background

Dental caries and gingivitis are two infectious diseases affecting a worldwide population and are the result of accumulation of the dental plaque, a polymicrobial biofilm on both tooth and gum surfaces [1,2]. Caries results from an acidic demineralisation of tooth hydroxyapatite (HA) produced by specific odontopathogenic bacteria (mainly *Streptococcus mutans*) in the presence of fermentable carbohydrates e.g. sucrose. The role of sucrose is fundamental in that it acts as substrate for a set of *S. mutans* glucosy-transferases (GTFs) which polymerise glucose from sucrose to a sticky exo-polysaccharide (called mutan). Mutan allows bacteria to tightly adhere to dental hydroxylapatite and, thus, to tooth surface. *S. mutans* has an efficient sugar metabolism and produces lactic acid (acidogenicity) with the additional capability of surviving in an acidic environment (aciduricity). The acid production is responsible for HA solubilisation, caries initiation and progression. Gingivitis (gum inflammation) results from accumulation of a heterogeneous subgingival plaque in which strict anaerobes dominate [3,4]. Overgrowth of strict anaerobes causes production of increased amounts of both bacterial toxins and catabolites. These are toxic for gingival cells and result in cell death and tissue inflammation. Thus, inhibition or reduction of dental plaque accumulation by various means is considered one of the best approaches to accomplish an effective prevention of diseases [1,2].

Consumption of foods and beverages rich in sugars coupled with poor oral hygiene are considered leading causes of dental plaque overgrowth and accumulation. During the past two decades several components of a variety of common foods (mainly vegetables) have been characterised by *in vitro* antibacterial and antiplaque assays and show potential anticaries and/or antigingivitis activities see for recent reviews [5,6].

In previous work we has shown that low-molecular-mass (LMM) fractions from either mushroom (*Lentinus edodes*) or red chicory (*Cichorium intybus*) homogenates (at sub-growth inhibitory concentration) interfere with binding of *S. mutans* (the main etiological agent of the dental caries) cells to HA and *Prevotella intermedia* (an example of periodontopathogenic bacteria) cells to gingival cells [7,8]. Additionally, biofilm formation of both odonto- and periodonto-pathogenic bacteria and detachment from preformed biofilms has been described for the compounds mentioned above [8]. Further purification of mushroom extract has been recently performed and a sub-fraction (i.e. # 5) has been identified as containing the majority of the biological activities [Papetti et al., manuscript submitted]. Beside these observations, the antimicrobial mode of action has been evaluated at the minimal inhibitory concentration, and an antibiotic-like mode of action have been described for the LMM fractions of both mushroom and chicory extracts. This activity strongly inhibited DNA synthesis and partially RNA synthesis with 50% reduction of protein synthesis in both *S. mutans* and *P. intermedia*[9,10]. As result of cell division (septum formation) inhibition, a certain degree of cell elongation has been observed in *S. mutans* (i.e. from ovoidal cells of the control to rod-like cells), while *P. intermedia* elongated from rods to form filaments. These morphogenetic effects are reminiscent of those obtained by treatment with antibiotics such as β-lactams or quinolones, two distinct families acting on different targets but having in common the inhibition of septum formation and, therefore the capability of filament formation [9,10].

The aim of this study was to characterise the bacterial receptors for the purified mushroom sub-fraction #5 in order to better elucidate the mode of action of these compounds. More precisely when interfering with bacterial adhesion to host surfaces or with bacteria-bacteria interactions when they are in the biofilm state. This is with a view to develop reliable tools for screening new natural compounds capable of binding (and inactivating) bacterial target(s). Candidate bacterial molecules to act as targets for these compounds are bacterial surface molecules. We considered cell wall associated proteins (CWPs), teichoic acid (TA) and lipoteichoic acid (LTA) of *S. mutans* and outer membrane proteins (OMPs) and lipopolysaccharide (LPS) of *P. intermedia*.

## Methods

### Strain used and growth conditions

*S. mutans* UA159 and *P. intermedia* ATCC 25611 were used throughout this study. *S. mutans* cells were grown in brain hearth infusion broth (BHIB) or on brain hearth infusion agar (BHIA) (Oxoid Ltd, Basingstoke, England). Cultures were incubated at 37°C in an atmosphere enriched with 5% CO_2_ as previously described [10]. *P. intermedia* was grown in BHIB to which 5 μg/ml haemin and 1 μg/ml vitamin K (Sigma-Aldrich Co, St. Louis, MO, USA) were added (BHIB + HK) or in Blood Agar (BA, Oxoid) plates. Cultures in both liquid and solid media were incubated at 37°C in an anaerobic chamber (Whitley DG 250 Anaerobic Workstation, Don Whitley Scientific, Shipley, UK) with an atmosphere composed of 85% nitrogen, 10% hydrogen and 5% CO_2_[9].

### Extraction of *S. mutans* cell wall associated proteins (CWPs)

CWPs were extracted with 8 M urea as previously described [11]. Briefly, 1 litre of an exponentially growing culture of *S. mutans* (O.D. _540 nm_ 0.7 O.U.) was rapidly chilled and cells collected by centrifugation (5,000 × g, 10 min) at 4°C. Cell pellet was extensively washed with 10 mM sodium phosphate buffer (pH 7.2), resuspended in 8 M urea extraction fluid and incubated for 1 h at RT. The extract was then dialysed against 10 mM sodium phosphate buffer (pH 6.5) to remove urea and proteins concentrated by 60% (saturation) ammonium sulphate precipitation and finally dialysed against 20 mM sodium phosphate buffer (pH 7.5) containing 1 mM phenylmethylsulfonyl fluoride. Protein concentration was determined by the BioRad kit (BioRad Laboratories srl, Segrate, Italy).

### Extraction of *S. mutans* TA

TA were extracted with 5% trichloroacetic acid (TCA) as previously described [12]. The pellet from one litre of an exponentially growing culture obtained as above was twice extracted with 5% TCA o/n at 4°C. Supernatants, after centrifugation at 18,000 × g at 4°C, were pooled and TA precipitated with 5 volumes (v/v) of absolute ethanol at 4°C for 16 h. Precipitated TA was collected by centrifugation at 18,000 × g at 4°C and resulting pellet washed twice with ethanol and finally with diethylethere. TA was resuspended in 2 ml of double distilled water, neutralized and concentration determined by estimating the organic phosphorus using the method of Chen et al. [13].

### Extraction of *S. mutans* LTA

LTA was extracted from de-acetylated bacteria with the hot phenol procedure essentially as described by Signoretto et al. [12]. Briefly, harvested bacteria from 1 litre culture were treated twice with chloroform-methanol (2:1 v/v) at RT for 2 h and then once with 45% aqueous phenol at 68°C for 45 min with stirring. Phenol was removed by dialysis against 0.1 M sodium acetate (pH 5.0). Nucleic acids were, then, degraded by extensive treatment with both DNase and RNase. Finally, additional phenol extraction and extensive dialysis against 20 mM sodium phosphate (pH 7.5) were performed to remove nuclease proteins and nucleic acid fragments. The resulting LTA was quantitated by measurement of phosphorus [13].

### Extraction of *P. intermedia* OMPs

OMPs were extracted after selective removal of inner membrane proteins as previously described [14]. An exponentially growing culture (1 litre, O.D._540 nm_ 0.3 O.U.) was rapidly chilled and collected by centrifugation as described above. Cells were disrupted by sonication, unbroken cells removed by low-speed (2,000 × g) centrifugation and, finally cell envelopes collected by ultracentrifugation (40,000 × g) for 60 min at 4°C. Inner membrane proteins were removed by treatment with 1% Sarkosyl (Sigma-Aldrich) and 1 mM phenylmethylsulfonyl fluoride for 1 hour at RT. After further ultracentrifugation, the resulting pellet was considered outer membrane. OMPs were solubilised with 0.5% *n*-octyl-β-D-thioglucoside in 20 mM sodium phosphate buffer (pH 7.5) and 1 mM phenylmethylsulfonyl fluoride for 2 h at RT. The supernatant after a further ultracentrifugation was extensively dialysed against 20 mM sodium phosphate buffer (pH7.5) to remove the *n*-octyl-β-D-thioglucoside.

### Extraction of *P. intermedia* LPS

LPS was extracted from whole cells using the procedure described by Eidhin & Mouton [15]. This method allowed water extraction of LPS at 100°C followed by digestion with proteinase K. Finally, proteinase K was removed by hot phenol treatment at 65°C and LPS lyophilized.

### LMM fraction of mushroom extract

The LMM fractions (< 5,000 Daltons) of shiitake mushroom (frozen shiitake mushroom *Lentinus edodes* was purchased from Asiago Food SpA, Veggiano, Padua, Italy) was prepared by ultrafiltration of the crude homogenate using the Vivaflow 200 system (Vivascience AG, Hannover, Germany) equipped with a membrane 5,000 MWCO PES for ultradiafiltration, as described elsewhere [7]. About 70% and 50% (w/w) of the components originally present in the crude mushroom homogenate was detected in the ultradiafiltrates. The ultradiafiltrates were sterilized using a 0.20 μm pore size membrane (Vivascience), then freeze-dried and stored up to 3 months at -80°C. Immediately before use, a sample was rehydrated with sterile distilled water to obtain a 10× solution and kept at 4°C for no longer than a week. The 1× concentration of the LMM fraction after reconstitution represents the original concentration in the food.

### Preparation and chemical characterization of sub-fraction #5 from the LMM fraction of mushroom extract

Sub-fraction # 5 was purified by gel filtration chromathography (GFC) from a LMM fraction of mushroom extract as previously described for *Cichorium intybus*[16]. Briefly: GFC analyses were carried out on an Agilent 1100 series liquid chromatography system (Agilent, Waldbronn, Germany) equipped with a diode array detector. The Agilent Chemstation software was used for HPLC system control and data processing. A Merck Superformance Universal glass cartridge system (300 mm × 10 mm) was used for GFC separation and the analyses were performed with a TSK gel Toyopearl HW-40 F (exclusion limits 100–10000 Da; Tosoh Corporation, Tokyo, Japan) with Millipore grade water as the mobile phase, at a flow rate of 0.5 mL/min. UV spectra were recorded in the 190–600 nm range, and chromatograms were acquired at 210 nm. Six fractions were collected and sub-fraction #5 corresponded to the compounds eluting at 154.3 ± 0.5 min.

The sub-fraction #5 was further separated and characterized by liquid chromatography with tandem mass spectrometry (LC–ESI/MS/MS). The analyses were performed with a Gemini C18 analytical column (150 × 2.0 mm, i.d., 5 lm; Phenomenex, Torrance, CA), connected to a Hypersil Gold C18 guard column (10 × 2.1 mm i.d., 5 lm; Phenomenex, Torrance, CA), with a binary mobile phase methanol/water acidified with 0.1% formic acid 5/95 (v/v) at a flow rate of 0.3 mL/min. Column and autosampler temperatures were held constant at 4°C. Chromatograms were recorded at 210 nm. Chemical characterization revealed that sub-fraction #5 contains 11 sub-sub-fractions mainly composed of quinic acid, uridine, adenosine, inosine, aconitic acid, oxalic acid and succinic acid.

### Binding of sub-fraction #5 of LMM fraction of mushroom extract to epoxy-activated Sepharose 6B

Epoxy-activated Sepharose 6B resin (Sepharose, Sigma-Aldrich) was chosen for the diversity of chemical groups of the ligands (hydroxyl, amino, thiol) that can be involved in resin coupling. In addition, resin has long hydrophilic spacer arm which make it particular suitable for immobilization of small molecules such as those included in sub-fraction # 5. Preparation of the resin for binding the sub-fraction # 5 as well as de-activation of the remaining active groups after coupling was performed according to manufacturers instructions. Finally, coupled resin was stored in 20 mM sodium phosphate buffer (pH 7.5) with 0.05% sodium azide. The amount of the phenolic compounds bound to the resin was determined by the Folin-Ciocalteu method (Sigma-Aldrich). The specificity of binding of the bacterial surface molecules was determined by preparing a resin sample which was de-activated prior binding the sub-fraction #5.

### Binding to and detachment from the conjugated resin of the specific bacterial macromolecules

*S. mutans* CWPs and *P. intermedia* OMPs were mixed with 1 g of sub-fraction #5 coupled resin in 30 mM sodium phosphate buffer containing 20 mM CaCl_2_ (pH 7.5) and 0.05% sodium azide at 37°C for 2 h. Then, unbound proteins were removed by extensive washing of the conjugated resin with 50 mM phosphate buffer (pH 7.5). Detachment of proteins that specifically bound sub-fraction #5 was performed by adding 2% SDS and heating at 100°C for 5 minutes. Finally, proteins were visualized by 10% SDS-PAGE [17], coloured by silver staining (Bio-Rad Laboratories).

*P. intermedia* LPS in lyophilized form was resuspended in 30 mM sodium phosphate buffer containing 20 mM CaCl_2_ (pH 7.5) and 0.05% sodium azide and put in contact with the coupled resin at 37°C for 2 h. Resin was extensively washed with the same buffer and bound LPS was detached by adding 2% SDS and heating at 100°C for 5 minutes. Finally, LPS was visualized by 12.5% SDS-PAGE [17].

*S. mutans* TA or LTA was resuspended in neutralised distilled water and put in contact with the coupled resin at 37°C for 2 h. Resin was extensively washed with distilled water and bound TA was finally detached with 5% potassium persulfate at 120°C for 30 min. The amounts of bound TA or LTA were determined with by measurement of phosphorus [13].

Three replicate bindings were performed with two distinct preparations for all the bacterial surface molecules tested with very similar results.

## Results

### Coupling of the mushroom sub-fraction #5 to the resin

Epoxy-activated Sepharose 6B was chosen because of its ability to bind covalently hydroxyl-groups of several compounds contained in sub-fraction #5 of LMM fraction of mushroom extract. The protocol allowed us to bind 30 μmoles of phenol sub-fraction per ml of drained resin. Furthermore, de-activation of the active groups of the resin prior binding sub-fraction #5 allowed determination of the specificity of the further binding of the bacterial surface molecules tested. In this case no amount of sub-fraction #5 was bound to the resin as evaluated by phenol determination and, consequently, no bacterial surface molecule was bound (data not shown).

### Identification of *S. mutans* surface macromolecules acting as receptors of mushroom subfraction #5

**Figure 1:**
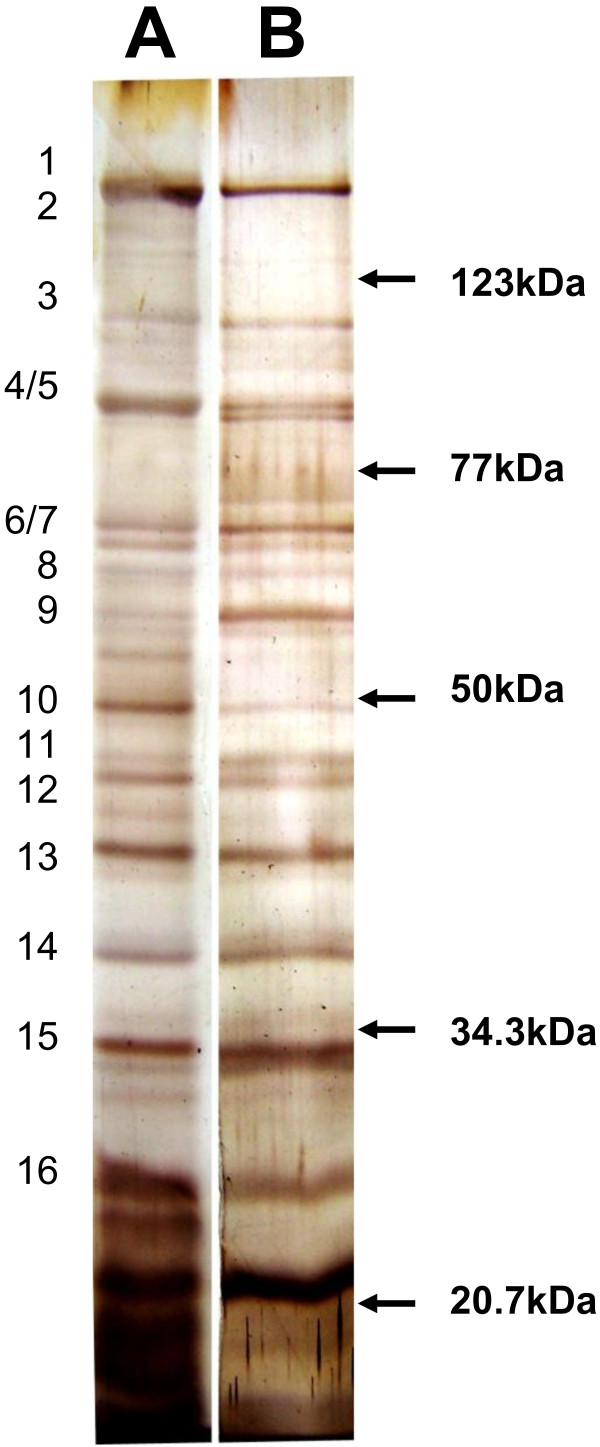
**Total cell wall proteins (CWPs) of *S. mutans *** **(A) and CWPs bound by sub-fraction #5 (B) separated by SDS- PAGE.**

Figure 1 shows a typical electropherogram of the *S. mutans* CWPs where 16 protein bands are visible. Based on their electrophoretic mobility, these CWPs have been identified after sequencing the entire *S. mutans* genome [18]. Figure 1 and Table 1 show that 15 of the 16 CWPs were capable of binding sub-fraction #5, with only FruA (M.W. 158,530) unbound.

**Table 1:** ***S. mutans *** **UA159 CWPs capable of binding sub-fraction #5**

**Band no.**^**a**^	**Approx. M.W.**^**b**^	**Binding to sub-fraction #5**	**Putative protein identification**^**c**^	**Sequence-deduced M.W.**^**c**^
1	170 KD	Yes	SpaP	168,841
2	155 KB	No	FruA	158,530
3	115 KD	Yes	AtlA	107,062
4	91 KD	Yes	dexA	94351
5	83 KD	Yes	GbpD	79,656
6	66 KD	Yes	GbpC	63,219
7	64 KD	Yes	SMU 1449	63,011
8	62 KD	Yes	GbpA	62,997
9	57 KD	Yes	Putative surface adhesin	57,392
10	50 KD	Yes	WapA	48,769
11	46 KD	Yes	GbpB	44,489
12	45 KD	Yes	GtfS	44,429
13	40.5 KD	Yes	RgpG	42,470
14	36 KD	Yes	GapC	35,937
15	33 KD	Yes	SloC	34,234
16	27 KD	Yes	StrA	27,342

^a^As specified in Figure 1.

^b^Determination based on electrophoretic mobility.

^c^According to the global sequence of *S. mutans* UA159 genome [18].

Table 2 shows the results of representative experiments of the binding of TA or LTA to sub-fraction #5: it shows that only TA had this capability, while LTA did not bind. Three binding attempts with each of two distinct LTA preparations were performed with very similar results.

**Table 2:** **Amount of *S. mutans *** **UA159 TA and LTA bound by sub-fraction #5**

**Macromolecule**	**Total (mg/L)**	**Bound (mg/L)**^**a**^	**Bound (%)**^**a**^
TA	28.9	23.8 ± 1.12	82.35 ± 4.49
LTA	38.3	0.11 ± 0.01	0.28 ± 0.002

^a^ Values are the means ± SD of three distinct binding experiments.

### Identification of *P. intermedia* surface molecules acting as receptors of mushroom subfraction #5

**Figure 2:**
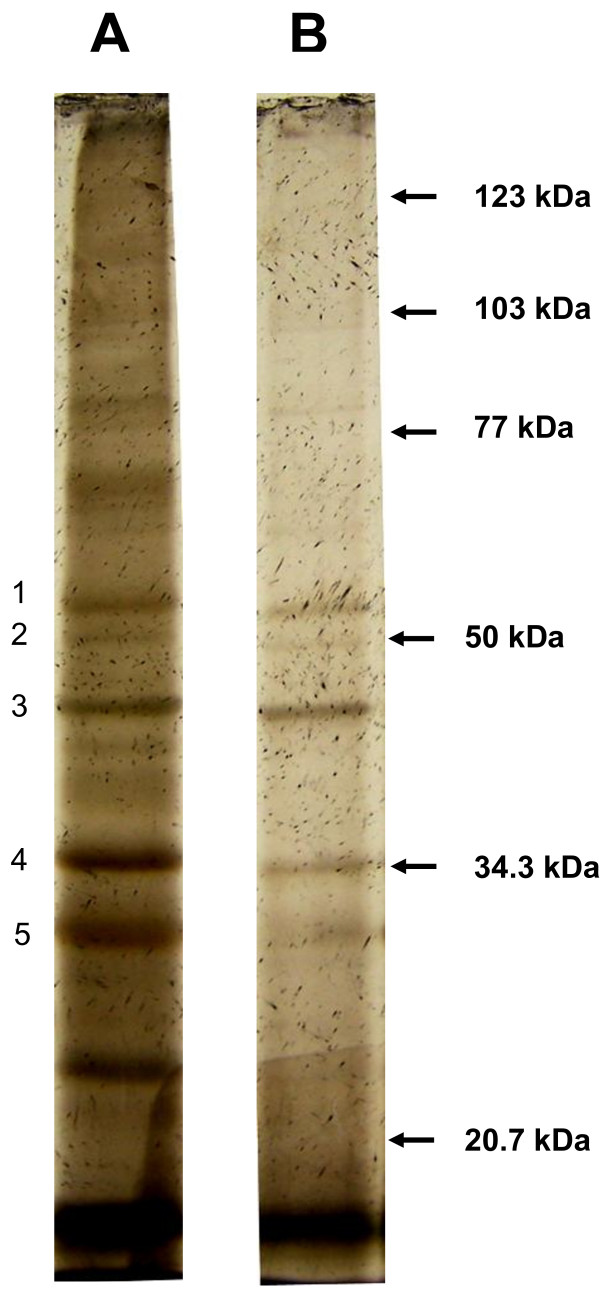
**Total outer membrane proteins (OMPs) of *P. intermedia *** **(A) and OMPs bound by sub-fraction #5 (B) separated by SDS- PAGE.**

Figure 2 shows a SDS-PAGE of the OMPs of *P. intermedia*. As expected a small number of protein bands (about a dozen) are shown in comparison to the whole proteome [19], however, when evaluated, only five proteins were detected capable of binding sub-fraction #5. Their approximate M.W. were 57, 51, 46, 36 and 33 KD.

**Figure 3:**
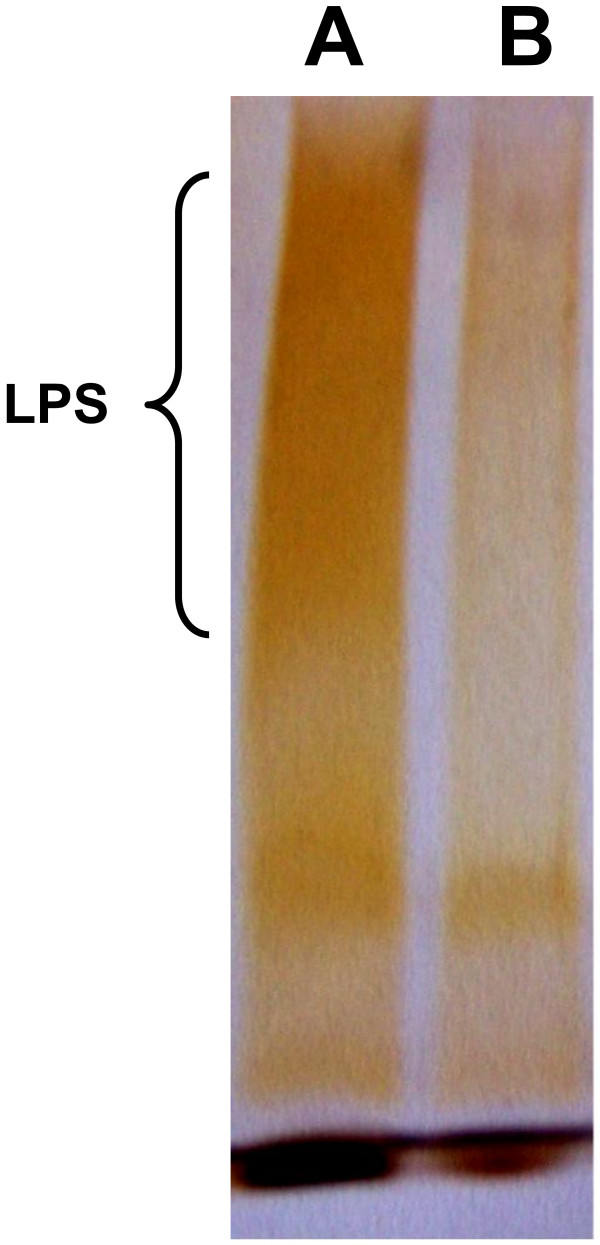
***P. intermedia *** **LPS (A) and LPS bound by sub-fraction #5 (B) visualized in SDS- PAGE.**

As far as the binding of LPS is concerned, Figure 3 shows that this envelope macromolecule was incapable of binding sub-fraction #5 in two distinct experiments with two separate preparations. It is worthy of note that *P. intermedia* ATCC 25611 is a “rough” strain (i.e. endowed with a LPS composed by lipid A plus core only), thus, in order to evaluate whether the absence of O chain could affect the binding to sub-fraction #5, we have used a commercially available *E. coli* O127:B8 (ATCC 12740) LPS (Sigma-Aldrich) purified from a smooth strain to try binding. In this case a positive result was obtained (data not shown).

## Discussion

Although foods and beverages and oral hygiene practices are still currently considered major contributors to oral infectious diseases such as caries and gingivitis/periodontitis, specific foods and beverages have been demonstrated to exert antimicrobial, antiadhesive and biofilm disgregating activities [5,6]. Among the numerous potentially active foods, fractions of them and identified bioactive compound(s), we have previously identified a LMM fraction of shiitake mushroom aqueous extract with promising properties [7,8]. This LMM fraction has been further fractionated and the best *in vitro* biological activities demonstrated to be associated with sub-fraction #5. [Papetti et al., manuscript in preparation].

*S. mutans* and *P. intermedia*, were chosen as appropriate representatives of disease; being an etiological agent of dental caries and a representative of the bacterial complex involved in gingivitis and later in progression to periodontitis, respectively. These were coupled with relevant assays which included inhibition of adhesion, inhibition of biofilm formation and biofilm disruption [16].

Because a main target of the bioactive compound(s) is the adhesion of bacteria to abiotic or biotic surfaces and with cell-cell interaction as in the case of biofilm formation, the aim of this work was to evaluate which bacterial surface molecules were targeted by the bioactive compounds. To do this, candidate bacterial surface compounds CWPs, TA and LTA in the case of *S. mutans* and OMPs and LPS of *P. intermedia* were prepared and used to characterise sub-fraction #5.

As far as *S. mutans* was concerned, 15 out 16 CWPs were bound by sub-fraction #5; this implies that the sub-fraction may play an important, but complex, role in inhibiting bacterial cell adhesion and biofilm formation. SpaP, the surface protein antigen A also known as protein I1, antigen B, Pac, SR and antigen I/II is a well characterised adhesin of *S. mutans*. Gene cloning and sequencing [20] has revealed a block of alanine-rich repeats and another of proline-rich repeats which are implicated in binding to salivary agglutinin glycoprotein gp340, a protein involved in saliva-mediated aggregation and adherence [21]. Importantly, antibody raised against SpaP blocked attachment of *S. mutans* to saliva-coated hydroxyapatite [22]. Sub-fraction #5 could act similarly. Wall associated protein WapA or Antigen III is released in the growth medium in a 29 KDa form although its gene encodes for a protein of 48,769 KDa. Knockout of *wapA* has an effect on other surface components, surface ultrastructure and biofilm formation [23]. WapE appears to alter cell surface and biofilm formation [18,24]. Glucan binding protein (Gbp) A, B, C and D are mediators of the sucrose-dependent adherence of *S. mutans* to polymers formed from sucrose. GbpA and GbpD contain a series of repeats (glucan binding domains) similar to those found in glucosyl transferases (GTFs) and, in addition to this, GbpD contains a lipase activity [25]. In vitro testing of knockouts of either GbpA or GbpD results in altered biofilm architecture suggesting a fundamental role in dental plaque structure [26]. GbpB and GbpC lack the repeats characteristic, sharing GbpB sequence homology with putative peptidoglycan hydrolases of other streptococci, thus hypothesizing a crucial role in cell wall turnover and stress response [27]. GbpC binds dextran tightly and, for this reason, is considered the major receptor involved in dextran-dependent aggregation, a mechanism involved again in biofilm architecture [26]. AtlA is a surface-associated protein that plays a critical role in surface biogenesis, biofilm formation, genetic competence and autolysis [28]. SloC, a cell wall associated component of the complex SloABC, is a solute-binding lipoprotein and a metal-dependent regulator which is involved in manganese and iron transport, thus regulating virulence gene expression of *S. mutans*[29,30]. SrtA or sortase is a transpeptidase that covalently links LPXTGX-containing surface proteins to the Gram-positive bacterial cell wall, included *S. mutans*. The *S. mutans SrtA* mutant is markedly less hydrophobic than wild-type, non adherent to hydroxyapatite, non aggregating in the presence of saliva and salivary agglutinin. Thus, sortase plays a crucial role in the surface-related properties by modulating the bacterial cell surface [31]. RgpG protein is involved in the synthesis of *S. mutans* rhamnose-glucose polysaccharide [32]. Although the mechanisms of cell surface polysaccharide synthesis are poorly characterized in Gram-positive bacteria, it is however clear that polysaccharides play crucial role in cell wall architecture and bacterial virulence [33]. DexA is a dextranase which can partially degrade glucan and, thus may affect *S. mutans* virulence [34]. GapC, an extracellular glyceraldehyde-3-phosphate, is involved in acid production from glucose [35]. Interestingly, inhibition of glycolysis by chlorexidine was demonstrated at GapC level [36].

It is known that polyphenols are capable of protein binding with high affinity and denaturation [37,38]. In this study we have shown for the first time that these polyphenol properties are expressed against proteins fundamentally involved in cell adhesion, biofilm formation and architecture, thus, justifying the previous in vitro observations.

In addition to the inhibition of biological functions of several CWPs involved in *S. mutans* adhesion and biofilm formation, TA has been shown to be bound by sub-fraction #5. This takes into account the biological role played by TA in cell wall structure of Gram-positive bacteria, an effect of disorganizing this fundamental bacterial structure may be suggested. On the contrary, purified LTA, at least in the experiments we performed, was unable to bind sub-fraction #5 and this may caused by the lipid moiety rather than the polysaccharide chain. It may be that in growing bacteria, since the lipid moiety is included in the cytoplasmic membrane and the polysaccharide component is included in the width of the cell wall, sub-fraction #5 may interact with this macromolecule.

If this were true, since LTA is involved in bacterial adhesion to both biotic and abiotic surfaces, further inhibition of this fundamental virulence property may occur.

As far as *P. intermedia* is concerned, we have evaluated the binding capability of the OMPs, showing that a few proteins interact with sub-fraction #5. OMPs play fundamental roles in Gram-negative bacteria functioning as a dynamic interface between the cell and the surrounding environment. Functions of these proteins include maintaining of cell structure, passive and active transport, adhesion to other cells, and binding a variety of substances [39]. Although studied to a lesser extent than those of Gram-negative facultative bacteria such as *E. coli*, similar functions can be attributed to the OMPs of Gram-negative anaerobes [19,40]. As in the case of *S. mutans*, binding sub-fraction #5 to surface proteins of *P. intermedia* may result in denaturation and inactivation of their physiological functions. This is compatible with the observed intererence of *P. intermedia* adhesion to gingival cells [8,41], biofilm formation and disgregation [8].

Capacity of binding sub-fraction #5 to *P. intermedia* LPS was also studied but in this case negative results were obtained. This event, however, may be justified by the fact that *P. intermedia* strain ATCC 25611 is a rough strain, i.e. containing a LPS composed only by lipid A and core but without a polysaccharide chain. Consequently, lipid alone is incapable of binding. Support of this statement comes from the observation that a commercially purchased LPS purified from an *E. coli* smooth strain, containing the polysaccharide O chain, is capable of binding sub-fraction #5. It is concluded, thus, that LPS may represent a target bacterial structure only in smooth strains. It is worthy of note to recall, however, that other major bacterial receptors may not be excluded and have been not tested.

Experiments performed more recently have shown that sub-fraction #5 contains 11 sub-sub-fractions mainly composed of quinic acid, uridine, adenosine, inosine, aconitic acid, oxalic acid and succinic acid [Papetti et al, manuscript in preparation] and biological activity relies mainly in quinic acid (QA). Very recently Papetti et al. [16] have shown that QA present in *Cychorium intybus* is one of the most active compounds capable of inhibiting virulence-related properties of oral pathogenic bacteria.

## Conclusion

This report suggests that, QA appears as a potential candidate to be used as active ingredient of products for daily oral hygiene. The advantage of natural molecules is that they comply with consumer’s demand for natural constituents of drugs and disinfectants.

Finally, the discovery of bacterial targets for the action of such compounds may allow us to set-up a simple laboratory method for selecting new natural compounds capable of binding and inhibiting the physiological function(s) of these bacterial surface molecules. This is an approach included in the so-called “antivirulence therapy” [6].

## Competing interests

The authors declare that there are no conflicts of interest in this study.

## Authors’ contributions

CS and PC equally contributed in experimental design, undertook data analysis and interpretation, and drafted the manuscript. AM, AB, and GB, carried out basic microbiology experiments. AP carried out fractionation of plant extracts. CP, EZ,PL, IO, DAS, JP, and MW are part of the Nutrident consortium and aided in the general experimental design. All authors approved the final manuscript.

## Pre-publication history

The pre-publication history for this paper can be accessed here:

http://www.biomedcentral.com/1472-6882/14/75/prepub
